# Reverse Transcription Polymerase Chain Reaction in Giant Unilamellar Vesicles

**DOI:** 10.1038/s41598-018-27547-2

**Published:** 2018-06-15

**Authors:** Mamiko Tsugane, Hiroaki Suzuki

**Affiliations:** 1 Department of Precision Mechanics, Faculty of Science and Engineering, Chuo University, 1-13-27 Kasuga, Bunkyo-ku, Tokyo, Japan; 2 Japan Society for the Promotion of Science (JSPS), 5-3-1 Kojimachi, Chiyoda-ku, Tokyo Japan

## Abstract

We assessed the applicability of giant unilamellar vesicles (GUVs) for RNA detection using *in vesicle* reverse transcription polymerase chain reaction (RT-PCR). We prepared GUVs that encapsulated one-pot RT-PCR reaction mixture including template RNA, primers, and Taqman probe, using water-in-oil emulsion transfer method. After thermal cycling, we analysed the GUVs that exhibited intense fluorescence signals, which represented the cDNA amplification. The detailed analysis of flow cytometry data demonstrated that rRNA and mRNA in the total RNA can be amplified from 10–100 copies in the GUVs with 5–10 μm diameter, although the fraction of reactable GUV was approximately 60% at most. Moreover, we report that the target RNA, which was directly transferred into the GUV reactors via membrane fusion, can be amplified and detected using *in vesicle* RT-PCR. These results suggest that the GUVs can be used as biomimetic reactors capable of performing PCR and RT-PCR, which are important in analytical and diagnostic applications with additional functions.

## Introduction

Biochemical and bioanalytical platforms using minute reaction volumes, often called as digital microfluidics have been powerful technologies to perform sensitive, rapid, and highly-parallel detection/screening using less amounts of chemical reagents. Currently, two platforms are commonly being used, namely microchambers (microwells)^1–3^ and microdroplets^4–6^. Microchambers provide solid-state boundary with well-defined volumes for biochemical reagents and enable precise quantification. The drawback of this solid container is its static nature owing to the difficulty in addition and extraction of reagents. Microdroplets are mainly produced using water droplets in immiscible solvent. Utilization of microdroplets has become a major approach as it provides increased throughput as well as dynamic reaction environment by enabling fusion and division^3^. Numerous assays such as enzyme reactions^7,8^, biochemical synthesis^2,9^, and cell responses^10–12^ were demonstrated in these small reactors. Among these assays, polymerase chain reaction (PCR) is one of the most widely used techniques in biotechnology. Therefore, the minute-volume PCR systems are being commercialised by using both the aforementioned platforms. As DNA is a stable molecule, the single copies of DNA distributed into a large number of small reaction volumes by dilution, can be readily amplified^3,13–15^. This technique is called digital counting and enables the absolute quantification of target DNA with low-abundance. The addition of the reverse transcription reaction prior to PCR (RT-PCR) enables the detection of target RNA, which is essential for gene expression analyses and RNA virus detection. Thanks to the development of one-pot RT-PCR system, the amplification of a single to a few tens of RNA molecules in microdroplets and microwells has been reported^16–20^.

The biological counterpart of the boundary of minute reaction environment is the lipid bilayer, which is ubiquitously present in living cells owing to its ability to form functional interfaces in an aqueous environment, adaptability to multiscales, and selective transportation of various chemical species and cargos. For engineering purposes, the utilization of lipid vesicles or liposomes has been mainly explored for drug delivery applications, where the chemical compounds, enzymes, or nucleic acids are encapsulated in nanometer-sized vesicles to administer them into the target cells^21,22^. Moreover, vesicles can be used as biochemical reactors^23,24^. In addition to simple enzymatic reactions, Overholzer *et al*.^25^ conducted PCR of 365 bp DNA in small (~185 nm, 3.3 aL) unilamellar vesicles (SUVs) obtained by the hydration and extrusion method. They detected the reaction product in a mere 0.1% of the vesicle population and concluded that the stochasticity in encapsulation of reaction components resulted in the ineffective amplification in small vesicles. More recently, Lee *et al*.^26^ amplified template DNA to increase the gene concentration (8.8 fold) by performing PCR in electrically neutral lipid vesicles (200 nm diameter) and these vesicles were used to transfect culture cells. As the vesicles are present in a water environment, reaction products can be directly administered into biological systems without extraction, unlike in the case of water droplets in oil.

Unlike small vesicles, giant vesicles (GVs) have sizes comparable to that of cells and water droplets in digital microfluidics (>1 μm in linear size and >1 fL in volume). Therefore, GVs can harbour complex biochemical reaction systems composed of multiple components. For example, spherical containers of 100 fL volume (~3 μm diameter) and 1 fL volume (~0.6 μm diameter) can contain 60 and 0.6 molecules, respectively, of a chemical species with 1 nM concentration (typical for enzymes and other macromolecules). Therefore, the large size of GVs increases the reaction efficiency by suppressing the stochasticity. To date, various multicomponent and multistep reactions such as the amplification of nucleic acids^27,28^ as well as translation and transcription^29–31^ were demonstrated in the GVs. However, most of these reactions were conducted at isothermal conditions at 37 °C. Although PCR is one of the most widely used techniques in biotechnology, its application in the GVs has been poorly explored. In fact, a very few studies have reported PCR in GVs. Recently, Shohda *et al*.^32^ performed PCR of 1229 bp DNA harbouring green fluorescent protein (GFP) gene in giant multilamellar vesicles (MLVs) obtained by the freeze-dried empty liposome method. They found that the reaction efficiency was ~20% and ~80% in 2.7 μm (10 fL) and 10 μm (~500 fL) vesicles, respectively. They concluded that the apparent low reaction efficiency might be attributed to the multilamellar and multivesicular structures of vesicles. The same group also conducted PCR in GVs formed by the natural swelling^33^ and freeze-dried rehydration methods^34^ in order to study the growth-division dynamics and its relevance to the DNA amplification. However, in these studies, they focused on the prebiotic behavior of “artificial cells” rather than the quantitative aspects of PCR.

We are developing a microreactor system using giant unilamellar vesicles (GUVs), which consists of single lipid bilayer similarly to the plasma membrane of cells, obtained by the water-in-oil (W/O) emulsion transfer method. This method was originally developed by Pautot *et al*.^35^, and can produce unilamellar vesicles of sizes up to ~100 μm in diameter^36^, and exhibits 100% encapsulation efficiency for a large range of molecular sizes and concentrations of reagents. Recently, several groups demonstrated the production of uniform-sized GUVs by transferring uniform-sized emulsions generated by the microfluidic devices^37–45^. Therefore, the W/O emulsion transfer method is suitable for constructing a well-defined reaction environment in the lipid bilayer. Moreover, GUVs can be used as a platform to obtain dynamic microreactors in which addition and extraction of reagents can be performed via the fusion and division of membranes^46–49^, similar to the vesicle trafficking which occurs in living cells. However, to the best of our knowledge, PCR in GUVs has not been reported till date.

In this study, we assessed the applicability of GUVs produced by using the W/O emulsion transfer method, to perform *in vesicle* RT-PCR which has practical importance for detection of transcripts. The RT-PCR mixture that contains the template mRNA, primer pair, and Taqman probe was encapsulated into GUVs to perform thermal cycling. The results of fluorescence microscopy images and flow cytometry analysis clearly indicated the amplification of cDNA which was synthesised from the template RNA. The serial dilution experiment of the template synthetic mRNA proved that the RT-PCR was successfully conducted using a small number of RNA molecules.

## Results

### RT-PCR in GUVs using total RNA

**Figure 1:**
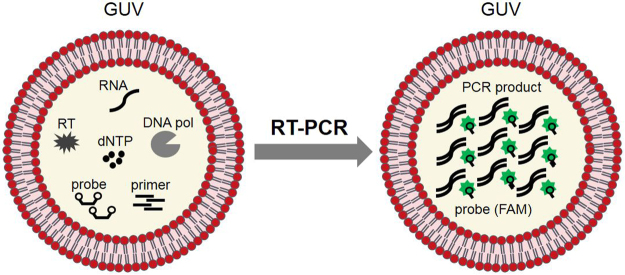
Schematic diagram of the RT-PCR system using giant unilamellar vesicles (GUV).

**Figure 2:**
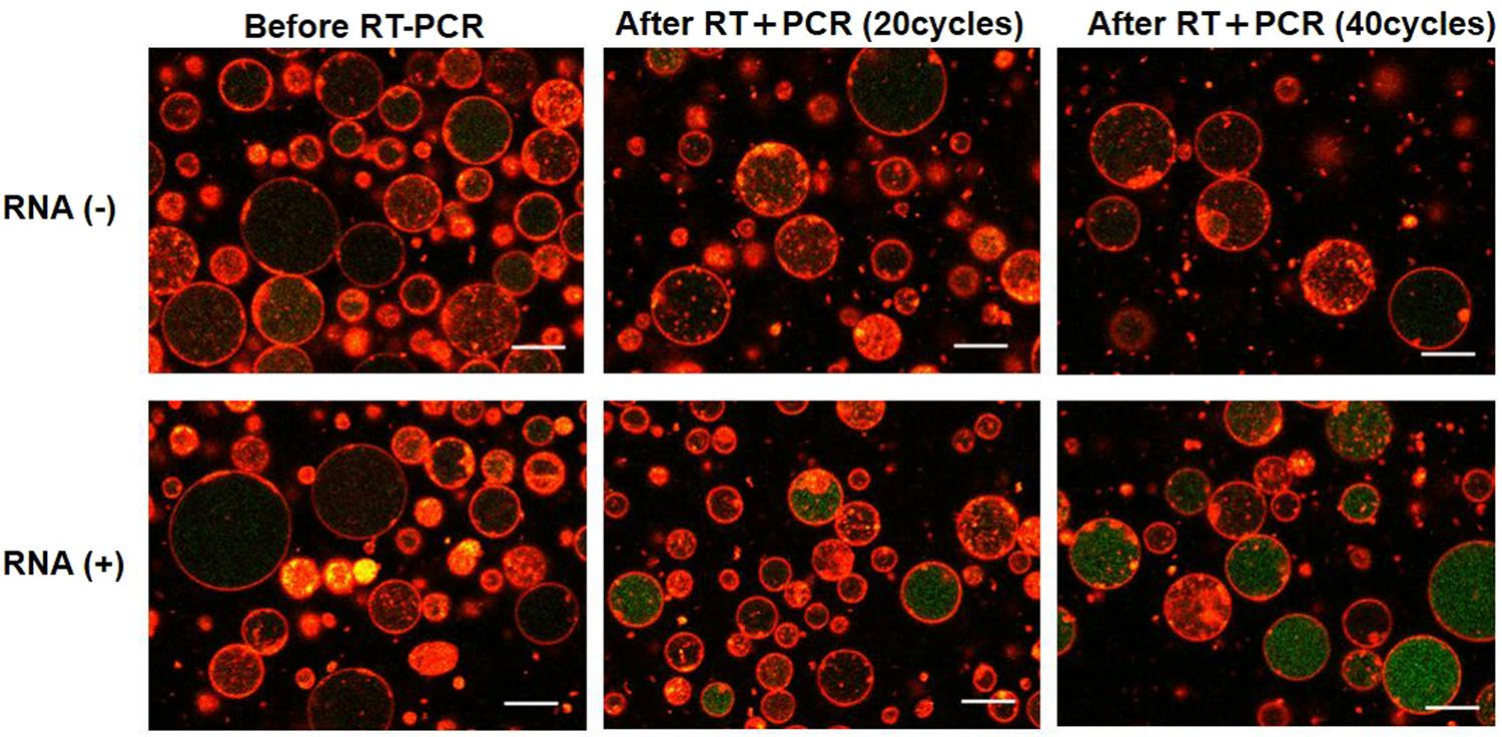
Fluorescence images of GUVs encapsulating RT-PCR mixture and rRNA probes were acquired using laser confocal microscope. The top and bottom images show the GUVs prior and subsequent to thermal cycling (20 and 40 cycles) in the presence and absence of template total RNA, respectively. Red: membrane stained with DiI and Green: Taqman probe (FAM). Scale bar = 10 μm.

Initially, we examined the conditions necessary to perform RT-PCR in GUVs using the total RNA extracted from human cell cultures. We prepared the one-pot reaction mixture including the total RNA, reverse transcriptase (RT), DNA polymerase (DNA pol), dNTPs, primer pair, Taqman probe conjugated with 6-carboxyfluorescein (FAM) reporter dye, and reaction buffer. Sucrose (200 mM) was supplemented into the reaction buffer to increase the relative density during centrifugation. This solution was emulsified in the oil phase (liquid paraffin) by vortexing with phospholipids as an emulsifier. Regarding lipids, we employed the mixture of 1-palmitoyl-2-oleoyl-*sn*-glycero-3-phosphocholine (POPC), 1-palmitoyl-2-oleoyl-*sn*-glycero-3-[phospho-rac-(1-glycerol)] (POPG), and cholesterol at 18∶2∶1 weight ratio, which was identical to that used in our previous reports^28,48–50^. In a test tube, this emulsion was mounted onto another water phase that will become the outer solution. This mixture was centrifuged to allow the water phase to pass through the lipid monolayer at the W/O interface to form lipid bilayer vesicles (Fig. 1). During RT-PCR, initially the cDNAs are synthesised from the RNA with reverse transcriptase and then these cDNAs are amplified using PCR. We used a commercial one-pot RT-PCR kit, which enables combining the reaction mixtures of the two-step process into a single preparation. During the *in vesicle* reactions performed in our previous experiments, the outer solution of GUVs was the same as the buffer of inner solution to avoid the generation of concentration or osmotic gradient. In the present experiment, as the buffer components of the commercial kit were unknown, we employed the common buffer solution to perform PCR (see Methods). Generally, the formation efficiency of GUVs in the hydration-based methods deteriorates as the ionic concentration of the buffer increases (the present buffer contains 50 mM KCl and 1.5 mM MgCl_2_), but it was possible to obtain unilamellar vesicles without notable negative effect by using the W/O emulsion transfer method (Fig. 2, left panels). We included a lipophilic dye 1,1′-Dioctadecyl-3,3,3′,3′-Tetramethylindocarbocyanine Perchlorate (DiI; DiIC18(3)) to visualize the membrane under a microscope. The fluorescence images demonstrated that the membrane was almost unilamellar without complex internal membrane structures. The size of GUVs ranged from one to several tens of micrometers in the fluorescence images. Importantly, GUVs were kept on ice to suppress the PCR reaction during all the preparation steps.

We quantified the amplification of target cDNA using the 5′ nuclease qPCR assay with Taqman probes. In this assay, an oligonucleotide with FAM dye at the 5′-end and a quencher at the 3′-end bound to the target sequence as a probe is cleaved by the endogenous 5′ nuclease activity of Taq DNA polymerase during each extension cycle. Therefore, as each quencher molecule gets separated, the FAM dye fluoresces to report the amplification of target DNA. In our first trial, we encapsulated the total RNA (at a final concentration of 2 ng/μL) extracted from the human culture cells as a template, a primer pair and a probe of eukaryotic 18S (chosen owing to 18S rRNA abundance in total RNA) along with the RT-PCR mixture.

Prior to performing the *in vesicle* reaction, we prepared GUVs that encapsulated the RT-PCR product and the reaction was completed in the test tube. The fluorescence images of these control GUVs exhibited intense green fluorescence from FAM solely within the internal volume, confirming that the amplification was detectable by microscopy (Supplementary Figure S1). Further, we conducted the RT reaction (42 °C for 5 min and 95 °C for 10 s) followed by the thermal cycling (95 °C for 5 s and 60 °C for 34 s for 20 or 40 cycles) using GUVs that encapsulated the unreacted mixture. The fluorescence intensity did not increase in the control GUVs without the total RNA as template, although primers and probes were present (Fig. 2, top panels). However, when the total RNA was included, intense green fluorescence was observed especially in the large GUVs (>5 μm diameter) (Fig. 2, bottom panels). The number of fluorescent GUVs increased as the number of thermal cycles increased from 20 to 40.

### Flow cytometry analysis

We performed flow cytometry analysis of GUV populations to quantify the fraction of fluorescent GUVs and its dependence on the GUV size (i.e. reaction volume). To this purpose, we included the fluorescence-tagged protein (transferrin from human serum, Alexa Fluor 647 Conjugate; TA647) in the reaction mixture as a volume marker instead of the membrane dye. We prepared 25 μL of GUV suspension that was subjected to thermal cycling in a test tube. Then, 10 μL of the resultant suspension was sampled and diluted 20 times using the outer solution and was analysed by flow cytometry. Four different signals i.e. forward scattering (FSC), side scattering (SSC), fluorescence intensities from FAM (*I*_FAM_), and TA647 (*I*_TA647_) were recorded.

**Figure 3:**
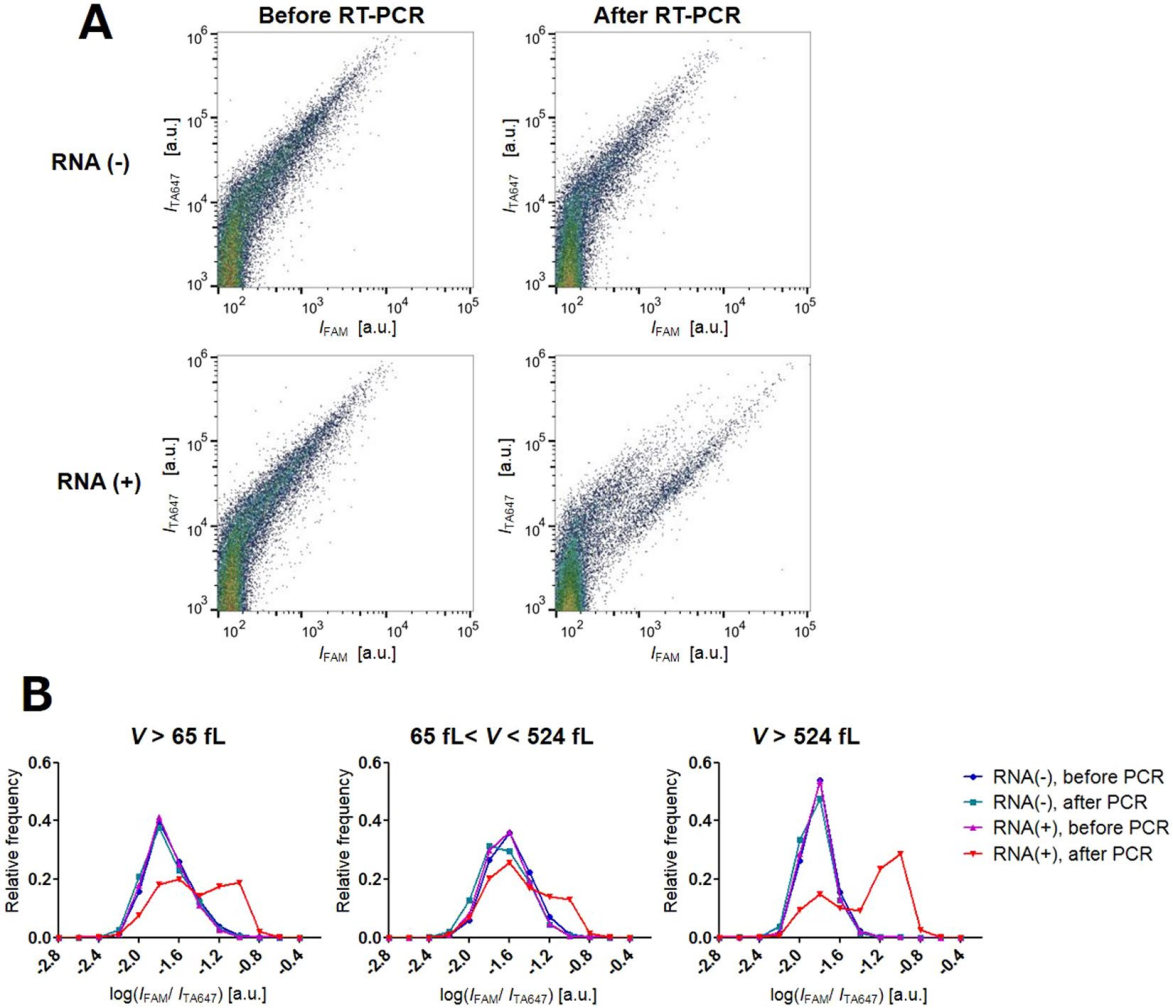
(**A**) Scatter plots of flow cytometric measurements of GUVs containing total RNA and 18S rRNA probes. Horizontal and vertical axes represent the fluorescence intensities of FAM (cDNA amplification) and Alexa 647 (volume marker), respectively. The four conditions correspond to those in Fig. 2 (40 cycles for RT-PCR). (**B**) Probability distribution plots of *I*_FAM_/*I*_TA647_ in logarithmic scale for GUVs with *V* > 65 fL. Plots of all the GUVs (left panel) are segregated and represented as 65 to 524 fL (middle panel) and above 524 fL (right panel) groups.

Then, we constructed the 2D scatter plot with *I*_FAM_ and *I*_TA647_ signals for ~7,000 GUVs (Fig. 3A). In these plots, the vertical axis (*I*_TA647_) was proportional to the reaction volume of GUV, and the horizontal axis (*I*_FAM_) was proportional to the amount of amplified DNA. Prior to RT-PCR reaction, the population was present along the line rising to the right with a slope of approximately 1, irrespective of the presence or absence of the template RNA (left panels in Fig. 3A). This result was similar to those of our previous reports^51–53^. Ideally, the *I*_FAM_ must be negligible, irrespective of the reaction volume. This linear dependence might be due to non-specific weak fluorescence from the uncleaved probe, and these plots are regarded as the background. After 40 thermal cycles, the distinct subpopulation with prominently high *I*_FAM_ appeared in the template RNA-positive GUVs (Fig. 3A, bottom right), whereas the overall distribution remained almost unchanged in the control GUVs without RNA (Fig. 3A, top right). As we look into the detail, there is a noticeable decrease in the relative density of large GUVs (*I*_TA647_ > 3 × 10^5^) and a corresponding increase in the small GUV population (*I*_TA647_ 10^4^–10^5^). We observed similar decrease in the signals with large FSC value, which indicates the size of particles (Supplementary Figure S2). These results indicated that the GUVs with relatively large size tend to break or rupture into small GUVs or to lose their internal contents including marker molecules by leakage.

Generally, there is a volume dependency in the biochemical reactions at small scales, mainly because of the stochastic encapsulation of low-concentration molecules and/or depletion of molecules onto/through the interface. The low amplification efficiency was also reported for PCR in small vesicles (<1 μm diameter)^25,32^. To elucidate this effect, we analysed the distribution of *I*_FAM_ after subdividing the populations into distinct ranges of the reaction volume (*V*). The reaction volume was estimated from the calibration curve that relates the *I*_TA647_ and the number of molecules obtained from the measurement of a set of calibration beads (Supplementary Figure S3; For more details, see Materials and Methods in Fujii *et al*.^50^). This volume conversion revealed that the number of GUVs with 5 × 10^2^ fL (~10 μm diameter) to 10^4^ fL (~30 μm diameter) decreased up to 70%, but the number of GUVs smaller than this range did not decrease after 40 thermal cycles (Supplementary Figure S4).

As seen in Fig. 3A (the right bottom panel), a substantial shift in *I*_FAM_ is observed in the GUVs with *I*_TA647_ > 5 × 10^3^ a.u. after RT-PCR. In the calibration curve, this intensity value using 1 μM Alexa 647 corresponds to *V* = 65 fL (volume of 5 μm sphere). Therefore, we analysed the *I*_FAM_ distribution after segregating the GUVs into 65 < *V* < 524 fL (volume of 10 μm sphere) and *V* > 524 fL groups, in addition to the sum of these populations (*V* > 65 fL). To eliminate the intrinsic linear dependence of *I*_FAM_ to *I*_TA647_, we plotted the histograms of *I*_FAM_ segregated based on that of Alexa 647 in logarithmic scale (log_10_ *I*_FAM_/*I*_TA647_) (Fig. 3B). This value corresponds to the internal concentration of fluorescent FAM probe. In all the three histograms, *I*_FAM_/*I*_TA647_ distributions of RNA-negative GUVs after RT-PCR were identical to those before RT-PCR (RNA negative as well as positive GUVs), indicating that non-specific amplifications did not occur in these minute compartments. Contrarily, using RNA-positive GUVs, bimodal peaks were observed in the whole population (Fig. 3B, left panel). Regarding subpopulations, a prominent distinction of the second peak with high *I*_FAM_/*I*_TA647_ values was observed in the large GUVs (*V* > 524 fL; Fig. 3B, right panel) compared to that in the small GUVs (65 < *V* < 524 fL; Fig. 3B, middle panel). In mammalian cells, the total RNA consists of 80% rRNA, 1–5% mRNA, and 10–15% tRNA in mass^54^. The present result suggests that it is possible to amplify the abundant rRNA in the total RNA at a concentration recommended by the manufacturer of RT-PCR kit (10 pg to 100 ng in 25 μL) in the GUVs with *V* > 524 fL.

**Figure 4:**
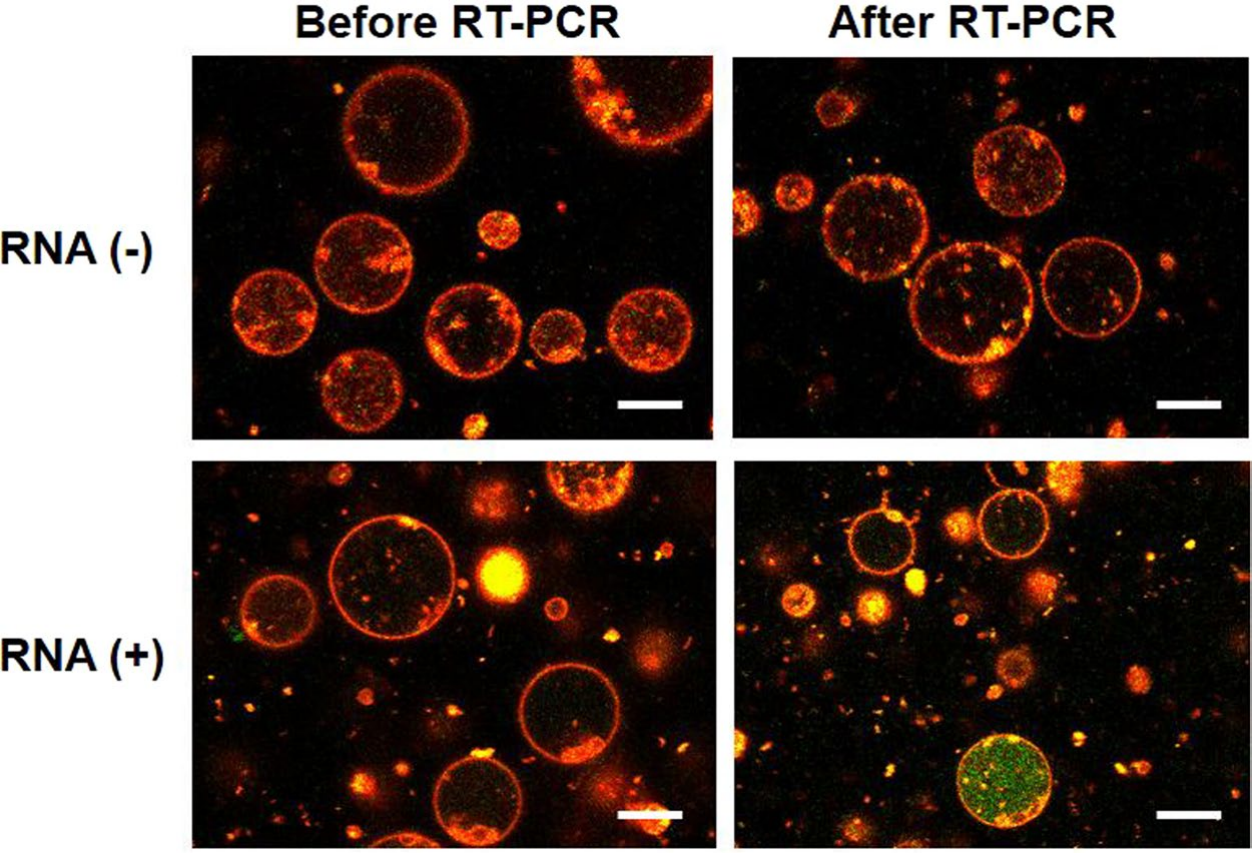
Fluorescence images of GUVs encapsulating RT-PCR mixture and β-actin probes. The top and bottom images show the contents of GUVs prior and subsequent to thermal cycling (40 cycles) in the presence or absence of template total RNA, respectively. Scale bar = 10 μm.

Further, in order to verify whether the mRNA present in low copy number among the total RNA could be amplified in the GUVs, we chose one of the housekeeping genes i.e. β-actin. We used the same concentration of the total RNA as that during rRNA amplification and included the primer pairs as well as probes for β-actin. The amplification curves of real-time PCR after RT reaction (Supplementary Figure S5) in tubes demonstrated that the copy number of β-actin mRNA was lower than that of rRNA. After encapsulating this reaction mixture into the GUVs, thermal cycling was performed. As depicted in the fluorescence images in Fig. 4, green fluorescence appeared solely in the GUV population containing the total RNA, but at lower frequency compared to that of rRNA probes (Fig. 2). In this case, the fraction of GUVs containing amplified-DNA was too low to be detected in flow cytometry analysis. The observed behaviour of the occurrence of *in vesicle* amplification should reflect the stochastic encapsulation of target mRNA molecules as a template.

### Efficiency of *in vesicle* RT-PCR using synthetic mRNA template

**Figure 5:**
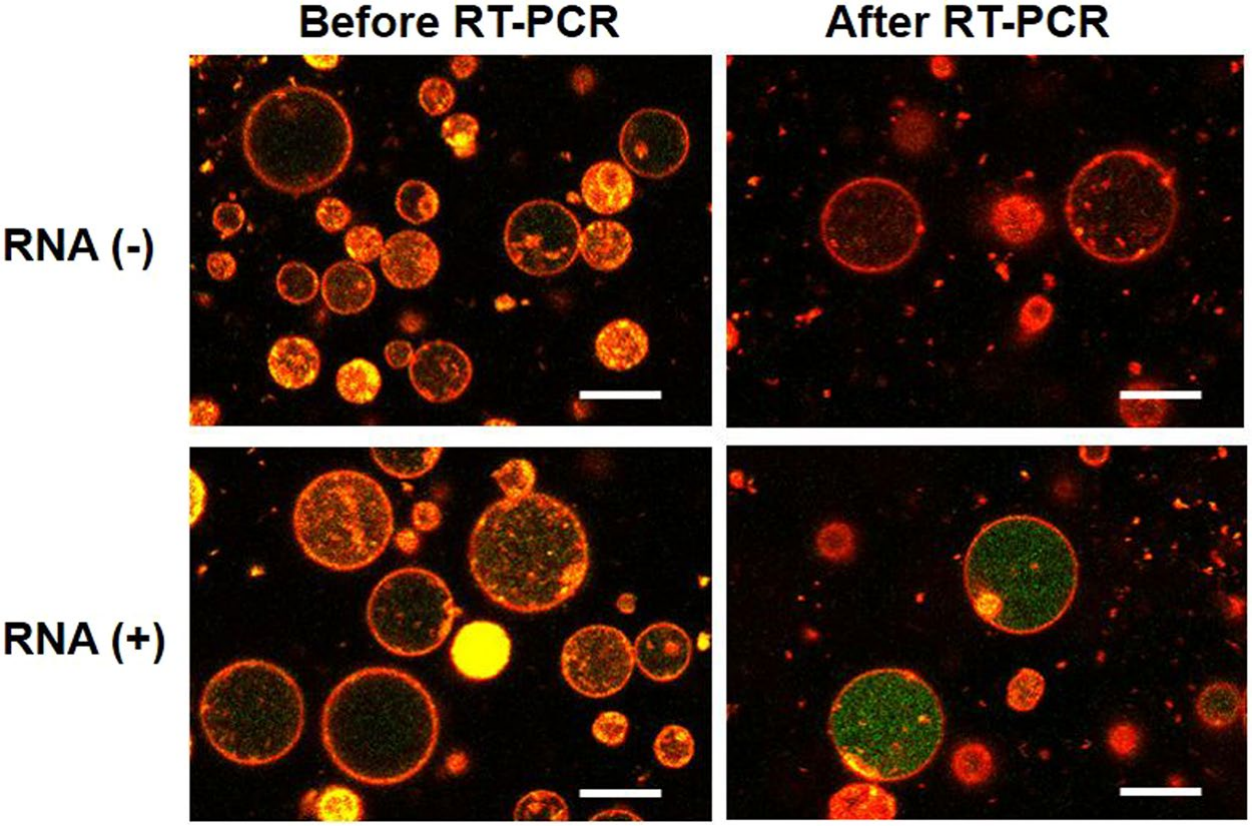
Fluorescence images of GUVs encapsulating RT-PCR mixture and β-actin probes. The top and bottom images show the contents of GUVs prior and subsequent to thermal cycling (40 cycles) in the presence or absence of synthetic mRNA (0.16 ng/mL), respectively. Scale bar = 10 μm.

In the *in vesicle* RT-PCR using the total RNA, the amplification of mRNA encapsulated in the GUVs was possible even at a low copy number. To quantitatively estimate the minimum detectable number of mRNA, we conducted *in vesicle* RT-PCR using synthetic mRNA encapsulated at a specific copy number. We prepared various RT-PCR mixtures containing 0, 1.6 × 10^−3^, 1.6 × 10^−2^, 0.16, and 1.6 ng/μL of β-actin mRNA as these dilutions correspond to 0, 0.1, 1, 10, and 100 copies of mRNA, respectively, in a spherical GUV with 5 μm diameter (65 fL). These mixtures were encapsulated into GUVs and subjected to thermal cycling. The fluorescence images of GUVs that encapsulated 0.16 ng/μL (10 copies/65 fL) mRNA prior and subsequent to RT-PCR are presented in Fig. 5. It was clear that the *I*_FAM_ was detected solely in the GUVs that encapsulated synthetic mRNA after 40 thermal cycles and the proportion of DNA-amplified GUVs increased with an increase in the concentration of mRNA (Supplementary Figure S6).

**Figure 6:**
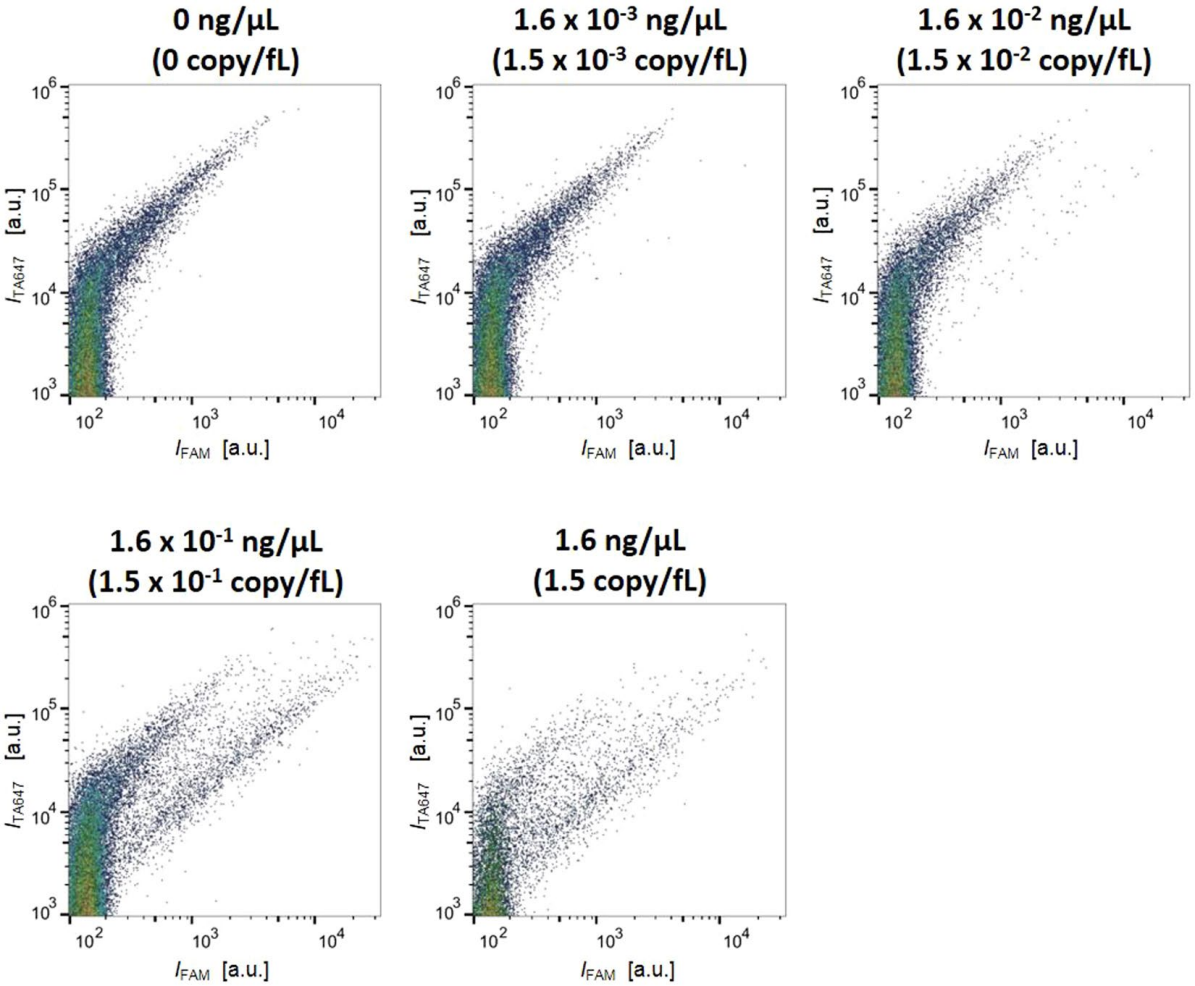
Scatter plots of flow cytometric measurements of GUVs obtained by performing amplification using synthetic β-actin mRNA template at various concentrations. Horizontal and vertical axes represent the fluorescence intensities of FAM (cDNA amplification) and Alexa 647 (volume marker), respectively.

**Figure 7:**
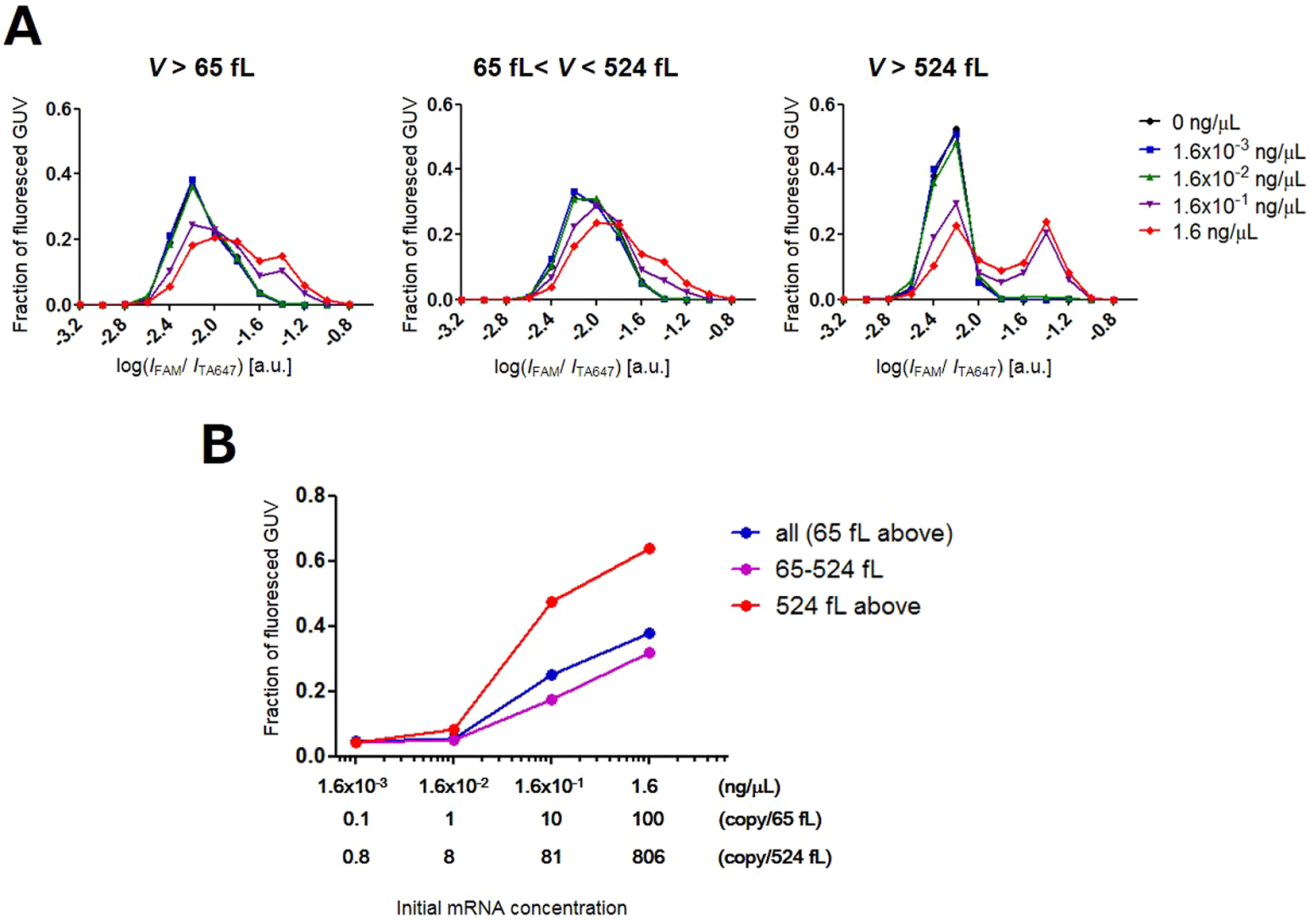
(**A**) Probability distribution plots of *I*_FAM_/*I*_TA647_ in logarithmic scale for GUVs with *V* > 65 fL after RT-PCR using various concentrations of mRNA. Plots of all the GUVs (left panel) are segregated and represented as 65 to 524 fL (middle panel) and above 524 fL (right panel) groups. (**B**) Plot representing the dependence of the relative frequency of reacted liposomes on the concentration of RNA templates.

Further, we analysed these GUV populations by using flow cytometry. In the absence of mRNA (0 ng/μL; Fig. 6), subpopulation deviation was not observed relative to the original population in the 2D scatter plot of *I*_FAM_ and *I*_TA647_. As the concentration of encapsulated mRNA increased, the number of GUVs exhibiting intense *I*_FAM_ gradually increased. At 1.6 × 10^−3^ and 1.6 × 10^−2^ ng/μL mRNA, a few scattered dots with high *I*_FAM_ values can be seen. Using >0.16 ng/μL mRNA, we observed a distinct subpopulation in numbers comparable to that of the original population. We analysed the *I*_FAM_/*I*_TA647_ distribution after subdividing the population into GUVs with 65 < *V* < 524 fL and *V* > 524 fL groups (Fig. 7A). Although the distributions of GUVs containing 1.6 × 10^−3^ and 1.6 × 10^−2^ ng/μL mRNA were similar to that with no mRNA, subpopulation with high value of *I*_FAM_/*I*_TA647_ appeared in the distributions of GUVs with 0.16 and 1.6 ng/μL mRNA. In these conditions, distinct bimodal peaks were observed in the GUV subpopulation with *V* > 524 fL (Fig. 7A, right panel). There is a slight upward shift in the frequency of GUVs with 1.6 × 10^−2^ ng/μL mRNA sample at the range of these second peaks for GUVs with *V* > 524 fL. Moreover, it is noteworthy that in the *V* > 524 fL subpopulation, the distributions of GUVs with 1.6 × 10^−3^ and 1.6 × 10^−2^ ng/μL mRNA were similar. The presence of concentration-insensitive population indicates that these GUVs have lost their amplification ability.

Based on the aforementioned analysis, we calculated the reaction efficiency i.e. the fraction of cDNA-amplified (fluorescent) GUVs. We determined the threshold of non-amplified GUVs by using the *I*_FAM_/*I*_TA647_ value of mRNA negative control experiment, below which 95% of GUVs after thermal cycling was included. The dependence of reaction efficiency on the template RNA concentration is presented in Fig. 7B. The number of cDNA-amplified GUVs was not increased in the *V* > 65 fL population with 0.1 copy/65 fL, while it increased slightly by 3% in the case of 1 copy/65 fL solely in the *V* > 524 fL population. In the case of 10 and 100 copies/65 fL, the overall reaction efficiency in the *V* > 65 fL population was 25 and 38%, respectively, whereas in the *V* > 524 fL population was 47 and 64%, respectively.

### RT-PCR in GUVs after electrofusion

One of the predictable advantages of lipid vesicle reactors compared to solid microchambers and W/O emulsion droplets is its structural similarity with the biological membrane. In living cells, lipid vesicles are ubiquitously present to encapsulate and transport bioactive molecules during the membrane trafficking. Analogous to these phenomena, we envisioned that the vesicle-based reactors can incorporate internal contents of the membrane-enveloped biological samples such as cells, organelles, and extra cellular vesicles via membrane fusion^46,55^. In this strategy, we expected that the membrane-encapsulated nucleic acids in intact biological samples could be directly transferred into the PCR or RT-PCR mixture in GUVs to perform detection and quantification, without involving the extraction processes. As a proof-of-concept experiment, we assessed whether RT-PCR could be performed after transferring the template RNA via the membrane fusion between GUVs.

**Figure 8:**
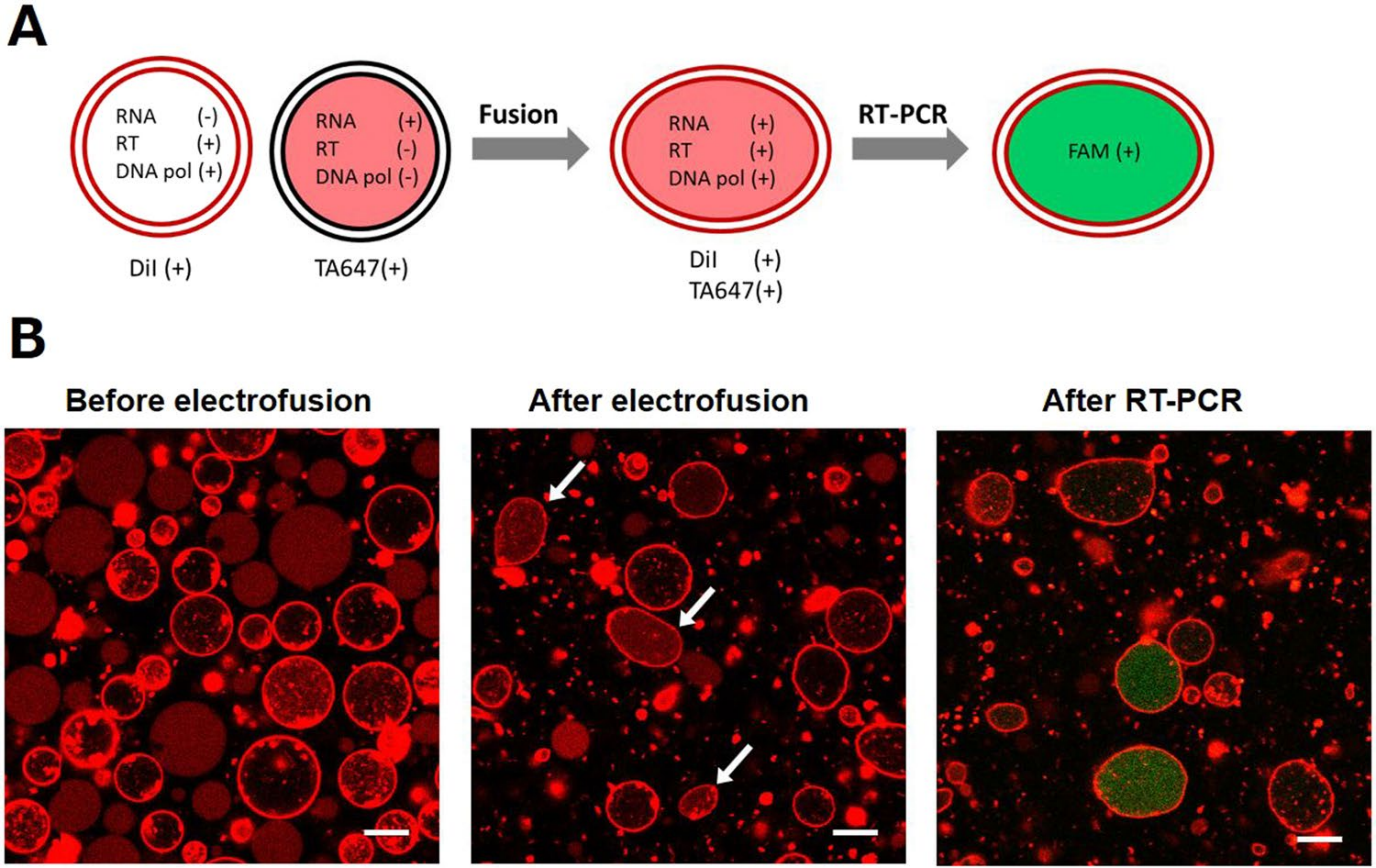
(**A**) Schematic representation of electrofusion and RT-PCR experiment. The two populations of GUVs that contain either the enzymes (RT and DNA pol) or template RNA were mixed into a single suspension. After electrofusion, RT-PCR thermal cycling (40 cycles) was conducted in order to amplify the cDNA present solely in the GUVs that contain both the components. (**B**) Fluorescence images show the GUVs prior and subsequent to electrofusion and after performing RT-PCR. Scale bar = 10 μm.

We prepared two GUV populations that contained either the reaction mixture (enzymes) or the template total RNA (4 ng/μL) (Fig. 8A). The primer pair and probe for rRNA were included in both the populations. The former population was marked using the membrane dye (DiI), while the latter was marked by the internal phase marker (TA647) to distinguish between these populations under the microscope. Here, 1,2-Dioleoyl-*sn*-glycero-3-phosphoethanolamine (DOPE) was supplemented in the lipid mixture as phosphatidylethanolamines with reverse cone-shaped molecular structure are reported to enhance the membrane fusion by accumulating at the stalk of fusion intermediate^56^. There was no noticeable difference in GUV formation process owing to the addition of 5% (w/w) DOPE. After mixing the suspensions of these two GUV populations at 1:1 volume ratio, the resultant suspension was introduced into the electrofusion cuvette with 1 mm electrode gap. Electrofusion of membranes was induced by applying short DC pulses (6 kV/cm, 60 μs, three times) after 1 MHz AC signal (15 V/cm, 15 s).

The fluorescence images of GUVs prior and subsequent to electrofusion, and after thermal cycling are presented in Fig. 8B. Prior to electrofusion, two distinct populations either DiI-stained membrane or TA647-stained internal volume were clearly visible at similar frequency (Fig. 8B, left panel). After electrofusion, GUVs containing membrane as well as internal markers appeared at a certain frequency (Fig. 8B, middle panel; indicated by arrows). These GUVs were mostly flaccid indicating that, when more than two spherical vesicles fused together, the fused vesicle exhibits larger membrane area than that of a sphere exhibiting identical total volume. After RT-PCR, we observed intense FAM fluorescence in a portion of flaccid GUVs as a result of cDNA amplification in fused GUVs (Fig. 8B, right panel).

## Discussion

In this study, we demonstrated that the reverse transcription and amplification of transcripts using the total RNA as well as synthetic mRNA could be conducted in GUVs. Although there was a significant decrease in the number of large-size GUVs (>10 μm diameter) during thermal cycling, we obtained a consistent amplification probability (fraction of fluorescent GUVs) depending on the copy number of encapsulated RNA templates within the remaining GUVs. Notably, the amplification probability in the GUVs > 5 μm in diameter was sufficiently high to be detected in the population analysis performed by flow cytometry. The amplification probability reached up to 64% in the GUVs with >10 μm diameter.

Assuming that 80% of the total RNA is rRNA, its number density in 2 ng/μL was calculated to be ~26 copies/65 fL using the total base-pair number of a ribosome (7,180 bp). Furthermore, the typical number of β-actin mRNAs per single human cell was reported to be ~1,000 copies^57^. Therefore, by assuming that the amount of total RNA and volume of single cell as 20 pg and 1 pL, respectively, the number density of β-actin transcripts in 2 ng/μL total RNA was calculated to be 6.5 × 10^−3^ copy/65 fL. In the experiment using the synthetic mRNA, we observed a population of fluoresced GUV prominently distinct compared to the control (RNA negative) population when the concentration of target RNA was >10 copies/65 fL. Therefore, the results obtained using the total RNA was consistent with those obtained using the synthetic mRNA. We might conclude that our system is able to detect the target RNA (10–100 copies) obtained from the total RNA in the GUVs with 5–10 μm diameter. Nowadays, reverse-transcription droplet digital PCR (RT-ddPCR; BioRad) kit is commercially available for the absolute counting of virions and transcripts. Thus, it is likely that the limit of detection (LOD) in GUV can be further improved by optimizing the reaction system.

However, the amplification probability did not reach 100% even at high template concentrations (rRNA and 10 or 100 copies/65 fL of synthetic mRNA). The amplification probability remained 64% in the large GUVs (*V* > 524 fL) with 100 copies/65 fL mRNA. It is probable that 40% of GUVs were non-reactive. Previously, Shohda *et al*.^32^ reported that 20% of the GUV population was non-reactive even in the template-rich condition. As they used vesicles obtained by freeze-dried empty liposome method, they concluded that the loss of efficiency was because of the substantially reduced apparent reaction volume owing to the subpartitioning in MLVs. In this study, we expected that the amplification probability might reach 100% after using GUV, as the occurrence of isothermal reactions such as gene expression was demonstrated in most of the GUVs in our previous report^53^. We presume that there might be leakage of small molecules (possibly dNTPs, primers, and probes), which exist solely inside the GUVs, because permeability of lipid bilayer membrane increases with temperature due to the loosened molecular packing of lipids^58,59^. As we defined that the large GUVs contain a high amount of TA647 (transferrin protein), large molecules such as enzymes must have remained in the GUVs. To confirm these hypotheses, we need to perform further experiments such as real-time observation under microscope during thermal cycling. Similar to the discovery of thermostable polymerase to perform PCR, improvement in the thermostability of the lipid membrane might be expected to provide a more reliable reaction environment. The use of bolalipids (tetraether lipids) found in Archaea bacteria^58,60^ or amphiphilic block copolymers might be one of the promising solutions.

Moreover, the amplification of target transcripts, which were directly incorporated via the membrane fusion, demonstrated a characteristic capability of the biomimetic reaction compartment. In conventional RT-PCR procedure, there is a possibility of degradation of active RNA during the extraction, purification, and preparation steps. In principle, our approach might overcome this problem to develop a highly sensitive detection system with minimum sample loss and simplified procedure. Although challenges remain in order to improve the efficiency, the fact presented by our study that PCR and RT-PCR can be conducted in the GUVs highlights the possibility of utilising these biomimetic compartments for developing nucleic acid detection system without the bulk extraction procedure.

## Methods

### RT-PCR solution

We used one-step real-time RT-PCR kit (RR064A; Takara Bio Inc., Shiga, Japan) supplemented with TaqMan probe and primers (Thermo Fisher Scientific Inc., Waltham, MA, USA) as the GUV inner solution. We employed the TaqMan probe (FAM/MGB) for rRNA and β-actin. Additionally, the reaction buffer accompanied to the kit was supplemented with 200 mM sucrose to obtain a density gradient with respect to the outer solution (200 mM glucose) during centrifugation. Regarding the template RNA, we used total RNA extracted from human culture cells (Agilent Technologies, Santa Clara, CA, USA) and synthetic mRNA of β-actin (Nippon Gene, Tokyo, Japan). The final concentration of total RNA was adjusted to 2 ng/μL and that of synthetic β-actin mRNA (1874 bp, MW 6.2 × 10^5^) was adjusted to 0, 1.6 × 10^−3^, 1.6 × 10^−2^, 0.16, and 1.6 ng/μL, which corresponds to 0, 0.1, 1, 10, 100 copies in GUV with 5 μm diameter (65 fL), respectively. To perform flow cytometry analysis, TA647 (Thermo Fisher Scientific Inc.) was included at 1 μM concentration in the inner solution.

### Preparation of GUVs

GUVs containing RT-PCR mixture were prepared using the W/O emulsion transfer method^35,50^. POPC, POPG (Avanti Polar Lipids, Alabaster, AL, USA), and cholesterol (Nacalai Tesque, Kyoto, Japan) lipids at 18:2:1 (w/w), or POPC, POPG, DOPE (Avanti Polar Lipids), and cholesterol at 17:2:1:1 (w/w) were dissolved in chloroform. Liquid paraffin (Wako Pure Chemical Industries, Osaka, Japan) was added to the aforementioned solution to adjust the final concentration of lipid mixture to 2.1 mg/mL. To observe under a microscope, a lipophilic dye DiI (0.05%, w/w; Thermo Fisher Scientific Inc.) was included in the lipid mixture. The liquid paraffin solution was warmed to 80 °C for 30 min to remove chloroform residue. After adding 25 μL of RT-PCR mixture, 400 μL liquid paraffin lipid solution was vortexed to obtain W/O emulsion. This emulsion was placed on 400 μL outer aqueous solution that contains 10 mM Tris HCl (pH 8.3), 1.5 mM MgCl_2_, 50 mM KCl, and 200 mM glucose in a test tube. This two-layered solution was centrifuged at 20,630 × *g* at 4 °C for 20 min to obtain GUVs precipitated at the bottom of tube as a pellet. This suspension of precipitated GUVs (~100 μL) was collected through a hole pierced at the bottom of test tube. Finally, after adding 400 μL of outer solution, GUV suspension was further centrifuged at 20,630 × *g* at 4 °C for 10 min and the supernatant was removed to obtain concentrated GUV suspension (~35 μL).

### Thermal cycling

GUV suspension (20 μL) placed in a PCR tube was subjected to PT-PCR using qPCR system (Mx3005P, Agilent Technologies). The thermal conditions were as follows: 42 °C for 5 min, 95 °C for 10 s, and [95 °C for 5 s and 60 °C for 34 s] × 40 cycles. The identical thermal conditions were also applied for 20 μL PCR solution without GUV to perform PCR in the test tube for the post-encapsulation experiment (Figure S1) and for checking the amplification curves (Figure S5).

### Microscopy experiments

Phase contrast and fluorescence imaging of GUVs was performed using confocal laser scanning microscope (LSM 700; Carl Zeiss, Jena, Germany) equipped with the 60×/NA 1.4 oil immersion objective. Fluorescence images were obtained using a 10 mW 488 nm laser for FAM (493–550 nm emission) and a 10 mW 555 nm laser for DiIC18(3) or TA647 (560–800 nm emission).

### Flow cytometry analysis

Quantitative analysis of RT-PCR in individual GUVs was conducted using Attune NxT flow cytometer (Thermo Fisher Scientific Inc.). The TaqMan probe and TA647 were excited by a 488 nm and 638 nm lasers, respectively, to obtain their fluorescent intensities. The number of TA647 molecules in an individual GUV were calculated from the fluorescence intensity of TA647 (*I*_TA647_) according to the calibration curve relative to the fluorescence intensity and number of Alexa 647 molecules attached to the calibration beads (Alexa Fluor 647 MESF calibration beads; Bangs Laboratories, Inc., IN, USA). Further, GUV volume was calculated assuming that 1 μM TA647 molecules were encapsulated at 100% efficiency. The final equation for conversion was *V* (fL) = (*I*_TA647_ × 9.1779)/602. Flow cytometry analysis was performed using FlowJo software (Tomy Digital Biology Co., Ltd., Tokyo, Japan).

### Electrofusion of GUVs

Two populations of GUVs were prepared using a lipid mixture of POPC, POPG, DOPE, and cholesterol (17:2:1:1, weight ratio) to perform the fusion assay. One population of GUVs was made to encapsulate the enzymes (RT and DNApol) without RNA, while the other population was made to encapsulate the total RNA and TA647 without enzymes. The primer pairs and probe for rRNA were included into both the populations. The membranes of the former GUVs were stained with DiI. To compensate for the dilution after fusion, the total RNA and enzymes were encapsulated at double the usual concentrations. One-to-one volume mixture (30 μL in total) of suspensions of these GUV populations was introduced into an electrofusion cuvette with 1 mm electrode gap, which was connected to an Electro Cell Fusion Generator (LF201; Nepa Gene Co., Ltd., Chiba, Japan). The alternative current was applied (15 V/cm, 15 s) to induce pearl chain alignment, and then, direct current short pulses (6 kV/cm, 60 μs, three times) were applied to induce the membrane fusion.

### Data availability

The datasets generated during and/or analysed during the current study are available from the corresponding author on reasonable request.

## Electronic supplementary material

Supplementary Information
