# Supplementary material for: Reverse Transcription Polymerase Chain Reaction in Giant Unilamellar Vesicles
Source: Sci Rep. 2018 Jun 15;8:9214. doi: 10.1038/s41598-018-27547-2 (PMC6003926; doi:10.1038/s41598-018-27547-2)
Supplement: Supplementary file 1 — Supplementary Information [file 41598_2018_27547_MOESM1_ESM.pdf]

Supplementary Information

# Reverse Transcription Polymerase Chain Reaction in Giant Unilamellar Vesicles

Mamiko Tsugane[1, 2] and Hiroaki Suzuki[1]*

[1] Department of Precision Mechanics, Faculty of Science and Engineering, Chuo University, Tokyo, Japan
[2] Japan Society for the Promotion of Science (JSPS), 5-3-1, Kojimachi, Chiyoda-ku, Tokyo, Japan

* Correspondence:
Prof. Hiroaki Suzuki
Chuo University
Faculty of Science and Engineering
Department of Precision Mechanics
1-13-27 Kasuga, Bunkyo-ku, Tokyo, 112-8551, JAPAN
suzuki@mech.chuo-u.ac.jp

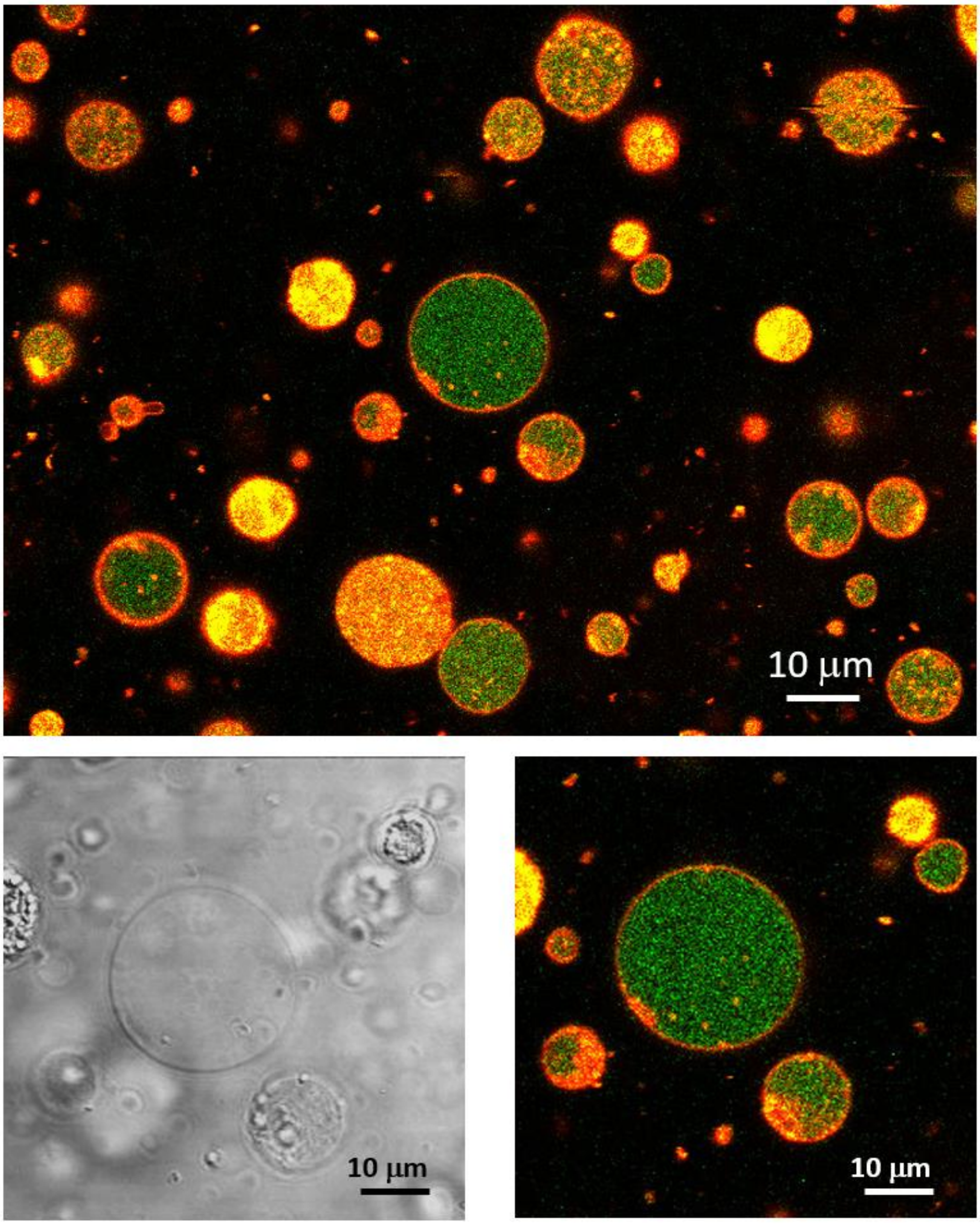


**Figure S1.** Fluorescence and bright field images of GUVs encapsulating RT-PCR products amplified in a test tube.

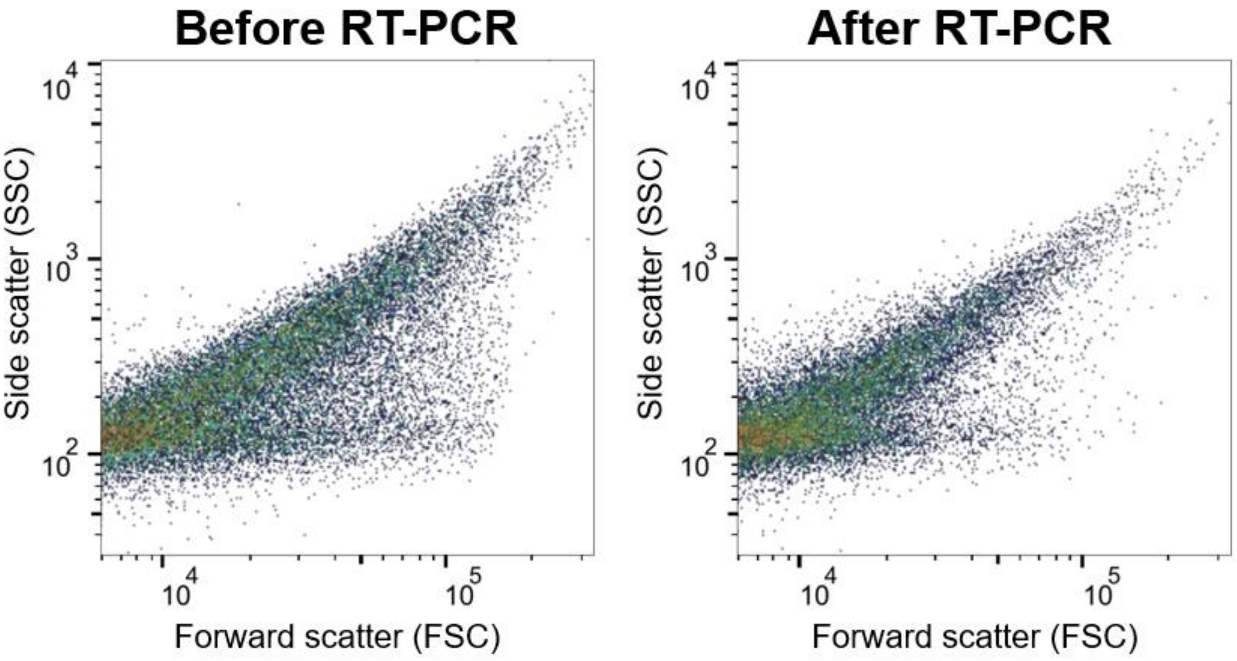


**Figure S2.** Scatter plots of flow cytometric measurements of GUVs prior (left panel) and subsequent (right panel) to RT-PCR thermal cycling. Horizontal and vertical axes represent forward scattering (FSC) and side scattering (SSC) signals, respectively.

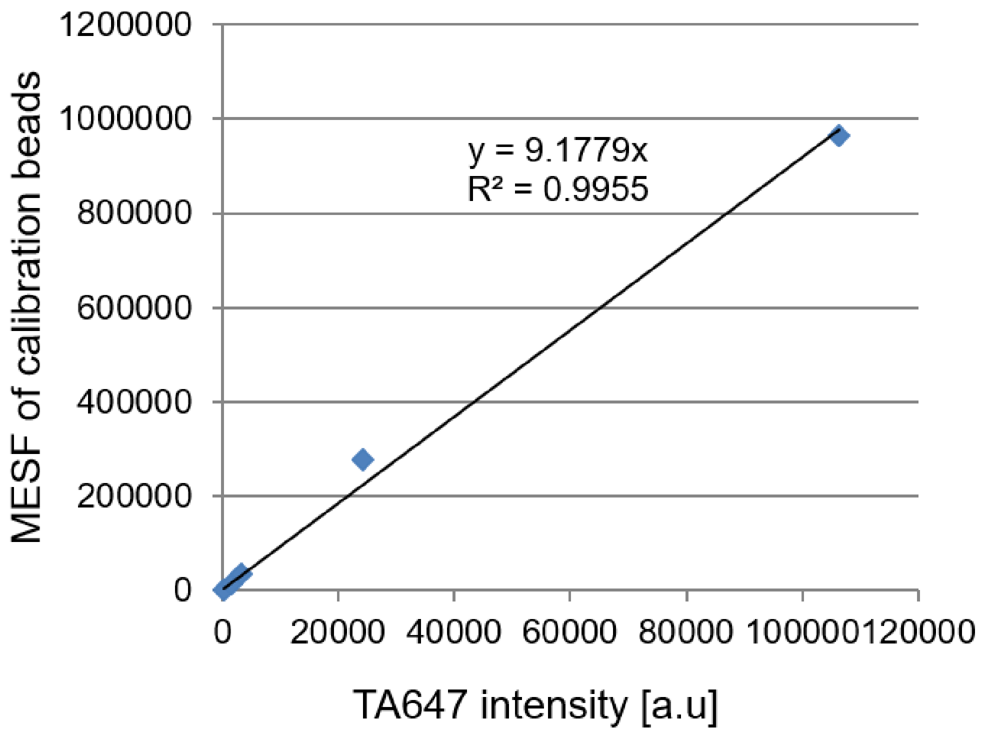


**Figure S3.** Calibration curve of vesicle volume (*V*) obtained using the measurements of calibration beads. The number of Alexa 647 molecules obtained from this curve was converted to vesicle volume based on the fact that a GUV contains 1 µM TA647. The final equation for conversion is *V* (fL) = (TA647 intensity × 9.1779)/602.

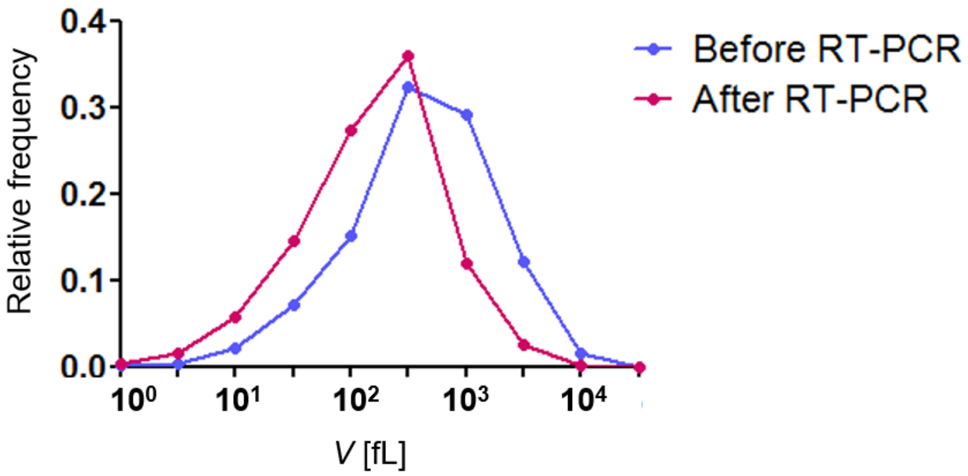


**Figure S4.** Distribution of GUV volume estimated from the fluorescence intensity of volume marker (TA647) prior and subsequent to thermal cycling.

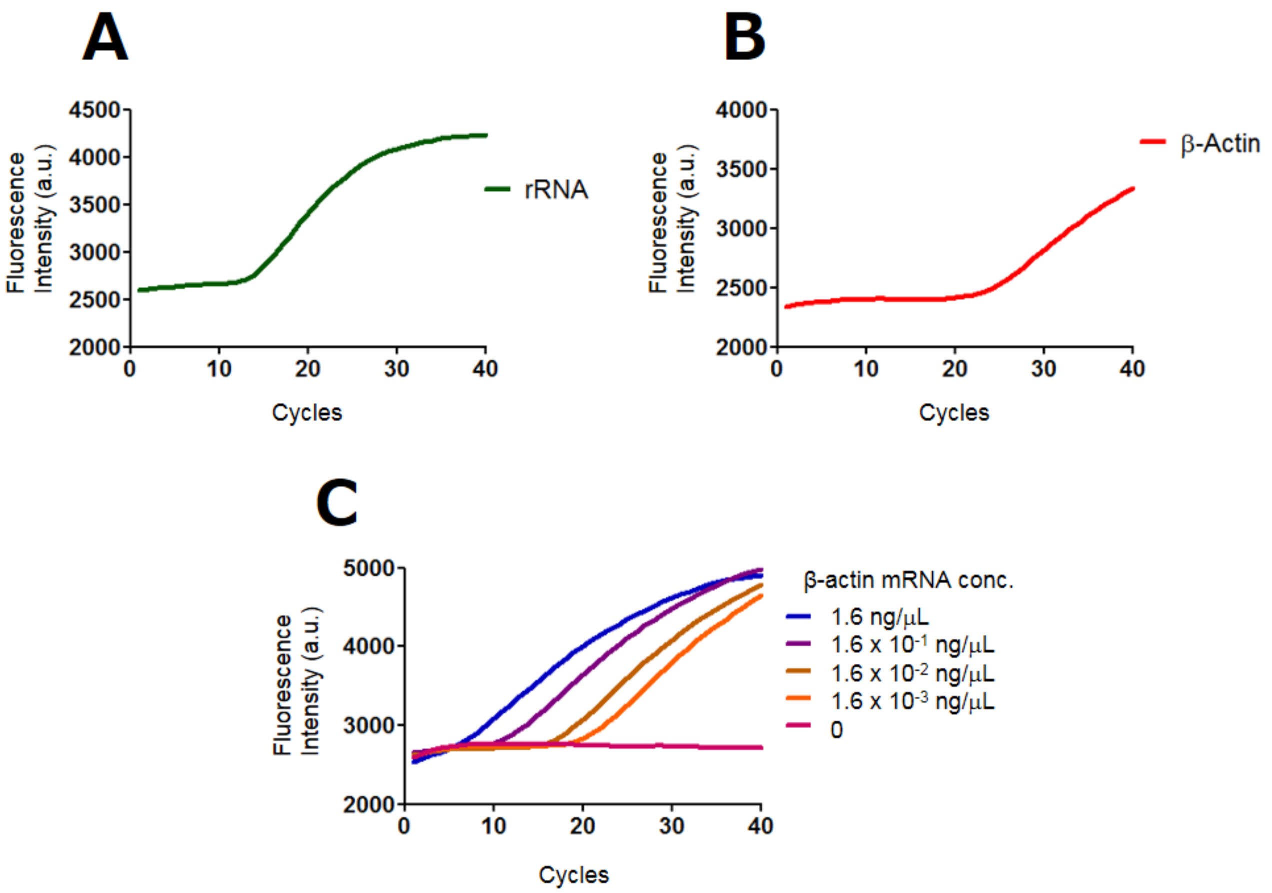


**Figure S5.** Amplification curves of RT-PCR in test tubes obtained using real-time qPCR apparatus. (**A**) Amplification of rRNA in the total RNA. (**B**) Amplification of β-actin mRNA in the total RNA. (**C**) Amplification of synthetic β-actin mRNA at various concentrations.

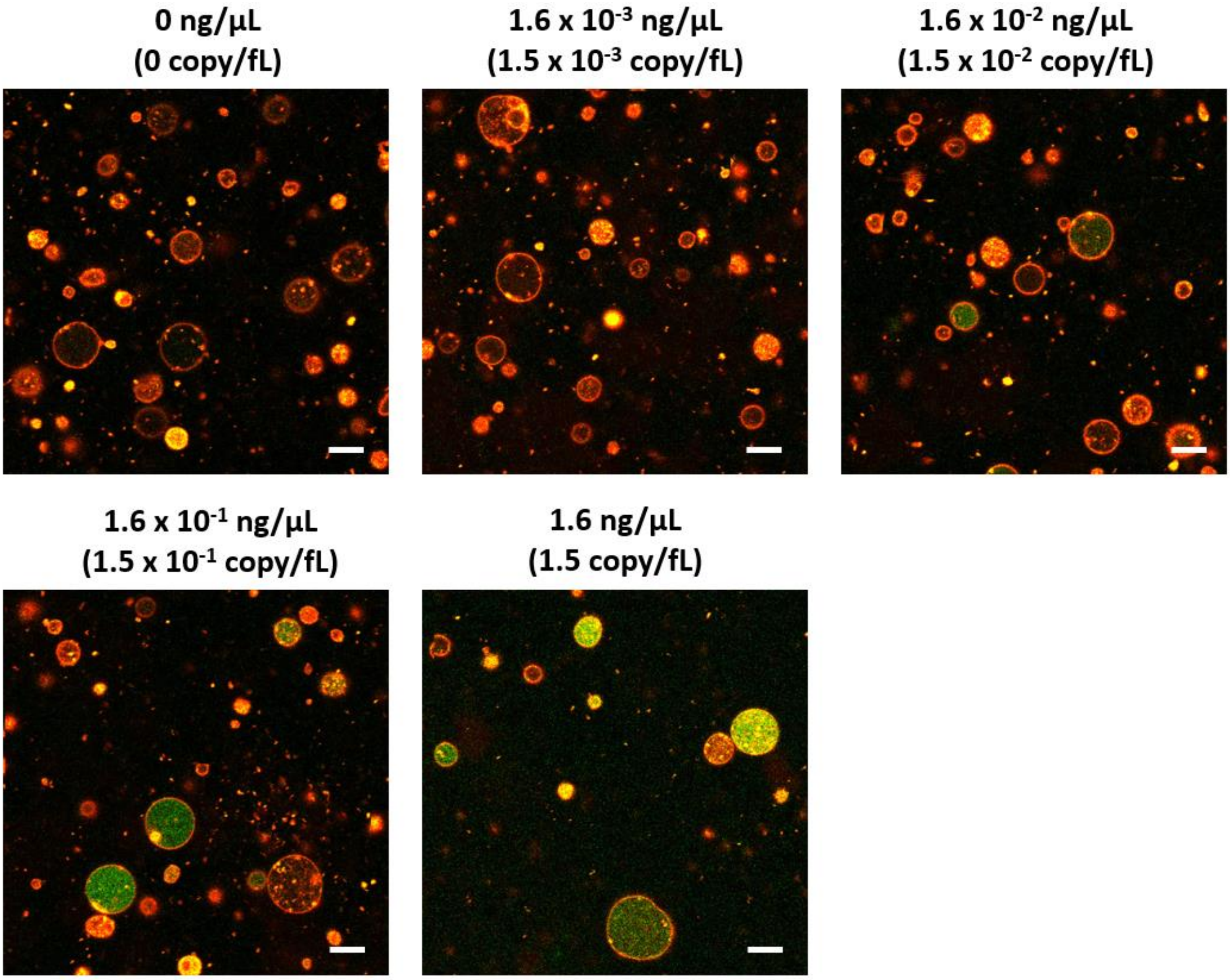


**Figure S6.** Fluorescence images of GUVs encapsulating RT-PCR mixture, β-actin probe, and synthetic β-actin mRNA at various initial concentrations after thermal cycling. Scale bar = 10 μm.
